# Iambic Production Advantage and Unbiased Recognition in Word Learning by Mandarin-Speaking Children with Cochlear Implants

**DOI:** 10.3390/bs16040491

**Published:** 2026-03-26

**Authors:** Xinyuan Shi, Jingjing Yang, Dandan Liang

**Affiliations:** 1 School of Chinese Language and Literature, Nanjing Normal University, Nanjing 210097, China; xinyuan.shi@njnu.edu.cn (X.S.);; 2 Interdisciplinary Research Center for Linguistic Sciences, University of Science and Technology of China, Hefei 230026, China

**Keywords:** word learning, stress pattern, Mandarin-speaking children, cochlear implants

## Abstract

This study examines whether Mandarin-speaking children with cochlear implants (CIs) exhibit challenges or advantages in learning novel words with overt trochaic versus iambic patterns. Mandarin full-tone words lack salient stress cues, whereas neutral-tone words exhibit a clear trochaic pattern. Given the unique prosody of Mandarin and CI users’ difficulty in acquiring the neutral tone, we predicted distinct effects of lexical stress on word production and recognition. Fifteen Mandarin-speaking preschoolers with CIs and 15 age-matched children with normal hearing (NH) learned 16 pairs of stress-contrasted novel words. A referent-naming task assessed stress production through pattern proportion, accuracy, and acoustic analysis, while a referent-matching task evaluated stress identification and word-referent mapping accuracy. In the naming task, children with CIs showed a preference for iambic words in both frequency and accuracy. They also produced longer second syllables in trochaic words than their NH peers. In the matching task, the CI group performed worse overall, although neither group showed a stress-specific effect. These results indicate that CI users struggle with syllable duration control in trochees. This difficulty reflects both an inability to shorten the unstressed syllable and the potential adoption of a final-syllable lengthening strategy linked to higher prosodic domains. The insensitivity to stress contrast in recognition may stem from the generally weak word-level stress cues in Mandarin.

## 1. Introduction

While children with cochlear implants (CIs) struggle with word learning (Houston et al., 2020) and vocabulary development (Ingvalson et al., 2023), their phonological processing deficits often exceed their morphosyntactic challenges (Nittrouer et al., 2018). These difficulties stem largely from the degraded speech signals provided by CI devices, especially fundamental frequency (F0) information (Meng et al., 2021), which limits auditory input compared to that of their normal-hearing (NH) peers. The present study examines the learning of disyllabic novel words with explicit trochaic and iambic stress patterns among Mandarin-speaking preschoolers with CIs. Word-level prosodic prominence, or lexical stress, refers to the relative prominence of syllables within a word. In nontonal languages, a stressed syllable is typically perceived as higher pitched, longer and louder (Hayes, 1995). While CI users acquiring nontonal languages remain sensitive to native stress patterns during word learning, Mandarin Chinese presents a unique typological case. In Mandarin, pitch primarily signals lexical tones rather than stress. As a result, while full-tone words lack overt stress patterns, words with a neutral tone exhibit a clear trochaic pattern (Xu, 2023). This linguistic specificity provides a novel case for investigating whether Mandarin-speaking children with CIs show distinct preferences or difficulties in learning novel words with trochaic versus iambic stress patterns.

### 1.1. Effect of Language-Predominant Stress Patterns on Word Learning in Children with CIs

Typically developing infants and children acquiring nontonal languages show early sensitivity to native stress patterns, which facilitates their word learning. This sensitivity begins in utero (Gonzalez-Gomez & Nazzi, 2012) and continues through infancy (Mehler et al., 1988). Newborns use rhythmic information to discriminate among language types (Nazzi et al., 1998). Consequently, word learning is shaped by phonological regularities of their native language, with prosody playing a role in early word representation (Segal et al., 2020). Young language learners internalize the native predominant stress pattern via speech input (Curtin, 2010; Gerken, 1994a, 1994b; Jusczyk et al., 1999), demonstrating language-specific biases in both word segmentation (Curtin et al., 2005; Johnson & Seidl, 2009; Polka et al., 2002) and word-referent associative learning (Graf Estes, 2014; Broś et al., 2021). However, the hypothesis of a universal trochaic preference (Allen & Hawkins, 1980) remains controversial. While some Hebrew-learning infants initially favor trochees despite the iambic nature of their native tongue (Adam & Bat-El, 2009), subsequent evidence suggests that trochaic versus iambic tendencies may be methodology-dependent. Specifically, trochaic biases being more prevalent in experimental settings, whereas iambic biases in naturalistic data (Kehoe, 2018).

Children with CIs struggle to perceive both word-level lexical stress and higher-level intonation (Most & Peled, 2007), while their production abilities vary across languages. For instance, Hebrew-speaking children with CIs produce various stress patterns with NH-like accuracy (Adi-Bensaid & Bat-El, 2004; Adi-Bensaid & Most, 2009), whereas English-speaking children with CIs show significant difficulties in spontaneous speech (Lenden & Flipsen, 2007). Pettinato et al. (2017) reported that during early babbling and first words production, English-learning infants with CIs employ weaker acoustic cues compared to their NH peers. Specifically, they showed overall smaller F0 and intensity distinctions, as well as reduced duration contrast between stressed and unstressed syllables.

Despite these perceptual and productive challenges, children with CIs can still access ambient lexical stress patterns and exhibit language-specific learning biases. For example, Hebrew-learning infants with CIs discriminate between trochees and iambs, favoring the predominant iambic pattern (Segal et al., 2016). Similarly, in trochaic languages such as English, Swedish, and Dutch, children with CIs show better imitation of trochaic words (Carter et al., 2002) or a greater tendency to omit word-initial unstressed syllables (Sundström et al., 2018). Moreover, native Dutch-speaking adults more easily identify stress contrasts in trochaic words produced by infants with CIs (De Clerck et al., 2019).

In summary, although children with CIs exhibit reduced stress sensitivity, they tend to follow the predominant stress patterns of their native language. Given that most existing evidence comes from nontonal languages, the present study extends this inquiry to Mandarin Chinese. We investigate whether Mandarin-speaking children with CIs can use lexical stress cues to facilitate their novel word learning.

### 1.2. Utilizing Novel Word Learning Tasks to Assess Word Learning Abilities in Children with CIs

Standardized assessments indicate that children with CIs generally exhibit word-learning deficits (Geers et al., 2003). To reduce the influence of linguistic familiarity and lexical density, studies frequently employ novel word tasks (Houston et al., 2005), where children are exposed to novel word-referent pairs and subsequently asked to identify or produce them immediately. Although children with CIs consistently underperform relative to their NH peers, they have the fundamental capacity to establish word-referent links (Davidson et al., 2014; Stelmachowicz et al., 2004).

However, children with CIs often find phonological production more challenging than comprehension (Pimperton & Walker, 2018). For instance, while Hebrew-learning infants with CIs can perceptually discriminate stress patterns (Segal et al., 2016), English-speaking CI users struggle to accurately produce them during repetition (Dillon et al., 2004). Evidence from Greek further highlights this discrepancy. Specifically, Greek-speaking children with CIs showed stress effects during word production but not recognition (Adamidou et al., 2023). Given that Greek is a language without a single predominant stress pattern, this asymmetry suggests distinct mechanisms underlying the recognition and production of lexical stress. That is, children with CIs may have difficulty assigning the correct stress pattern due to articulatory constraints or phonological deficits.

In summary, while infants and children with CIs possess the fundamental capacity to map novel words to referents, they generally demonstrate deficits in word learning, particularly in the accurate production of phonological forms. Like Greek, Mandarin Chinese lacks a single, overtly predominant stress pattern across its full tone vocabulary. This typological similarity raises the question of whether lexical stress influences novel word learning in Mandarin-speaking children with CIs, and whether such an effect manifests differently in word production versus recognition.

### 1.3. Lexical Tones, Neutral Tone and Lexical Stress in Mandarin Chinese

Mandarin Chinese is a tonal language where pitch primarily distinguishes lexical meaning through four canonical tones, i.e., T1 (high level), T2 (rising), T3 (dipping or low level), and T4 (falling) (e.g., *ma1* “mother”, *ma2* “hemp”, *ma3* “horse”, and *ma4* “scold”, where italics indicate pinyin forms and numbers indicate tone categories in Mandarin). in addition, the system includes a neutral tone (T0), which is characterized by a shorter duration and a pitch contour determined by the preceding syllable (Tang et al., 2018). While most Mandarin disyllabic words consist of two full-tone syllables, certain pairs contrast solely via stress patterns. For instance, *dong1 xi1* “east and west” contains two full-tone syllables, whereas *dong1 xi0* “thing” contains one full-tone syllable and one weak syllable with a neutral tone. Although such word pairs account for only 0.72% of dictionary entries (W. Li, 1981), they represent the most overt trochaic pattern in Mandarin.

Disyllabic full-tone words are common in Mandarin Chinese, and they can fit the disyllabic foot template if possible (Huang & Duanmu, 2013). For these words, lexical stress is perceptually subtle (Xu, 2023), though acoustically, the second syllable often exhibits longer duration and a more complete pitch contour (Lin et al., 1984). Previous studies have proposed that the Mandarin disyllabic foot is theoretically trochaic (Duanmu, 2014; Hsieh, 2021; Wang & Feng, 2006). Evidence for this stress pattern includes constraints on trisyllabic compound formation, where adding a stressed morpheme before a disyllabic word is often restricted to avoid stress clashes (Duanmu, 2014). Empirical support stems from coordinate compounds with irreversible syllable orders (Hsieh, 2021) and colloquial shifts where full-tone words reduce to trochaic neutral-tone forms (Wang & Feng, 2006).

For disyllabic words containing neutral tones, the neutral-tone syllable always occupies the right position; hence, these words clearly exhibit an overt trochaic pattern. Unlike English, where pitch is a primary correlate of stress (Hayes, 1995), Mandarin primarily uses pitch for lexical tones. Consequently, shorter duration and lower intensity are two distinctive properties of neutral-tone syllables, whereas pitch still plays a role in neutral tone identification (A. Li & Fan, 2015). Mandarin-speaking children with CIs face challenges in accurately producing neutral-tone words, which correspond to trochees (Tang et al., 2019; Yu et al., 2023). Specifically, except some familiar kinship reduplications, these children often fail to achieve adult-like acoustic realization, typically exhibiting a reduced F0 range for lexical tones and excessively long durations for neutral-tone syllables.

The unique prosodic property of Mandarin raises an interesting question: do overt stress patterns influence word learning in Mandarin-speaking children with CIs. More specifically, will these children be facilitated by a covert, theoretical trochaic pattern when learning novel words with experimentally imposed stress? Or will the acoustic challenges inherent to CI users inhibit their acquisition of trochaic patterns despite their theoretical prevalence in the native language?

### 1.4. The Current Study

The present study investigates the learnability of trochaic and iambic novel words in Mandarin-speaking preschoolers with CIs. Given that linguistic and perceptual skills in this population develop under conditions of early auditory deprivation, identifying their learning biases is essential for optimizing language interventions for children with severe-to-profound hearing loss. To evaluate word-referent associative learning, this study employs a novel word learning paradigm consisting of two tasks. The referent-naming task evaluates word production, examining whether children can accurately articulate the learned stress patterns. The referent-matching task assesses word recognition by requiring children to identify aurally presented novel words and match them with their corresponding referents.

Our hypotheses are grounded in the unique prosodic properties of Mandarin and the documented acoustic challenges faced by CI users. In Mandarin, overt trochaic patterns are limited to neutral-tone words, which are significantly difficult for children with CIs. We hypothesize that children with CIs will demonstrate lower overall performance and distinct learning patterns compared to their NH peers. First, we hypothesize that children with CIs would exhibit a production advantage for iambic words and face difficulty with trochaic ones. Because children with CIs tend to lengthen the second syllables within neutral-tone words, we expect this tendency to facilitate the production of iambic words, where the second syllable is inherently stressed and thus naturally longer. Conversely, we hypothesize that children with NH will show no systematic bias toward either stress pattern. Their normal auditory experience enables them to develop stable and abstract phonological representations of full tone and neutral-tone words. Their solid phonological skills should allow them to process and produce both trochaic and iambic novel words with comparable proficiency.

## 2. Materials and Methods

### 2.1. Participants

Two groups of children participated in the current study: the CI group and the NH group. The CI group consisted of 15 4- to 6-year-old prelingually deafened children with CIs (3 females; *M* = 4;9 [years; months], *SD* = 8 months) (see Table 1). These children were diagnosed with severe-to-profound congenital hearing loss before the age of three; they had a mean unaided pure tone average hearing loss ≥ 85 dB; they had been implanted before the age of five, ranging from 15 to 52 months (*M* = 2;8, *SD* = 11 months); and their CI experience ranged from 18 to 37 months (*M* = 2;0, *SD* = 6 months). Nonverbal IQ was assessed using the Wechsler Intelligence Scale for Children, and each participant with CIs received a score greater than 105 (*M* = 116.93, *SD* = 7.83). The NH group included 15 Mandarin-speaking NH children who were matched with the participants with CIs for gender (3 females), age (*M* = 4;9, *SD* = 8 months) and nonverbal IQ (*M* = 112.46, *SD* = 8.39). Independent sample t tests revealed that there was no significant group difference in age, *t*(28) = −0.47, *p* = 0.963, or nonverbal IQ, *t*(28) = 1.51, *p* = 0.143. All participants came from monolingual Mandarin-speaking homes, and they were neurologically and physically stable, with no history of social anxiety or mood disorders. The experimental procedure was explained to all participants’ parents and teachers, and permission was obtained from both parents of each participant.

### 2.2. Stimulus Construction

The experiment constructed 32 (16 disyllables × 2 stress patterns) novel words based on 16 real words in Mandarin. The real words were selected using the following procedure. First, we selected 96 disyllabic real words that exhausted all 16 combinations of the four lexical tones, with six words for each tone combination. Second, 15 teachers of children with CIs and 15 parents of preschool NH children participated in a familiarity assessment. Each real word was judged on a 7-point scale ranging from 7 (very familiar and frequently used) to 1 (very unfamiliar and never used). For each tone combination, the word with the highest familiarity value was selected to make sure that the selected words were familiar to preschoolers, yielding a total of 16 disyllabic real words. The mean familiarity value was 6.12 (*SD* = 0.56).

The novel words were constructed by manipulating the 32 syllables from the 16 disyllabic real words (see Table 2). First, each novel word was a combination of two syllables from the 32 syllables. The 16 recombined disyllables did not form tonal contrasts with any real word. Second, 16 pairs of novel words were constructed by contrasting stress patterns: in half of the cases, the first syllable was stressed, and in the other half, the second syllable was stressed.

These 16 pairs of novel words were recorded by a native Mandarin female speaker. The stress patterns associated with the recordings of the novel words were assessed by 15 undergraduate students majoring in linguistics. The recording of a novel word was retained only when more than 95% of judgments on its stress pattern matched the pattern produced by the female speaker.

Thirty-two pictures of referents differing in shape and color were selected. Each pair of pictures was selected to correspond to one pair of novel words: one corresponded to the referent of a novel trochaic word, and the other corresponded to the referent of a novel iambic word. None of the referents was familiar to the children. That is, each was an item that the children had never seen before, could not identify, and were unable to name. These referents were selected from online sources or adapted from previous studies (e.g., Davidson et al., 2014). To ensure that the referents were indeed unfamiliar, all items were pre-evaluated by 10 adults and 10 children who did not participate in the main experiment. Only those objects that could not be named or recognized by any of the raters were retained for the formal task.

### 2.3. Procedure

The experiment was performed using PowerPoint on a laptop in a quiet room. The children’s responses were recorded by Audition 1.5. The participants were seated approximately 1 m from the laptop screen. The aurally presented materials were delivered through a loudspeaker at approximately 65 dB SPL. The loudspeaker was placed at the same horizontal level as the seating position.

**Figure 1:**
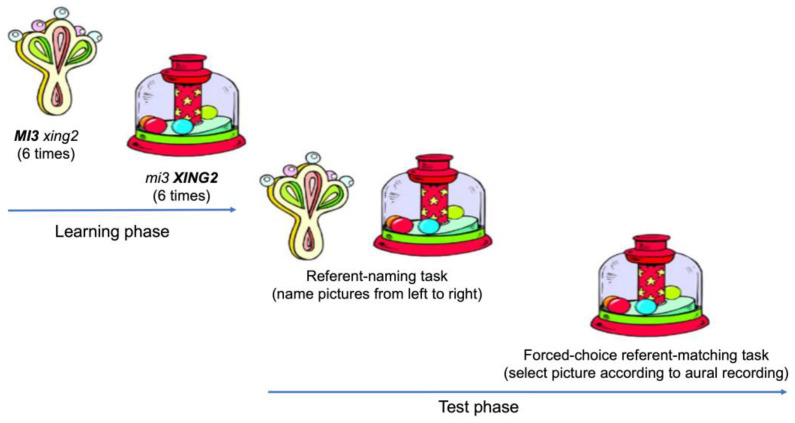
Procedure of each trail. Bold face marks the stressed syllables imposed in the novel words.

The participants completed a short training session to familiarize themselves with the procedure and understand the tasks before the main experiment. The training session and main experimental session had the same structure. The training session included five pairs of commonly used real words and their corresponding pictures. The main experimental session contained 16 trials in which 16 pairs of novel words were tested. As demonstrated in Figure 1, each trial consisted of a learning phase followed by a test phase. In the learning phase, the participant learned the two novel words one by one. A picture of one referent appeared on the computer screen, and the corresponding digital recording of the novel word was played. The participant learned the novel word by repeating the name of the referent every time after the recording ended. The recording was played six times; hence, the children repeated the word six times. After that, the other novel word was learned by the participant in the same way. In the test phase, a referent-naming task and a referent-matching task were conducted. In the naming task, the pictures corresponding to the first and second learned novel words were presented on the computer screen in a left-to-right sequence from the participant’s perspective. The participant was asked to name the left picture and then the right picture. The question for the naming task, *Zhe4 shi4 shen2me0 ya0?* “What is this?”, was asked by the experimenter. In the matching task, the two pictures remained on the screen, and the experimenter played one of the digitally recorded novel words and repeated the target word by asking the question *Na3 ge4 shi4 X?* “Which one is X?” (X here stands for the novel word). The participant was required to select the picture that corresponded to the novel word. No feedback was provided after the participants responded.

The presentation order of the trials was pseudorandomized to avoid a recency effect (see Table 3). In total, each participant produced 16 trochaic words and 16 iambic words in the naming task, and the participant gave eight responses for the trochaic words and eight responses for the iambic words in the matching task.

### 2.4. Data Collection and Analysis

For the referent-naming task, the stress pattern of each response was assessed by three phonetically trained Mandarin-speaking graduate students. They were asked to categorize the responses as *zuo3zhong4* “trochaic”, *you4zhong4* “iambic” or *deng3zhong4* “equal prominence”. The assessment of stress patterns took place in a quiet room, and recordings were presented via Cool Edit Pro V2.1. The stress pattern of a response was confirmed when all three native speakers gave the same judgment; otherwise, the response was excluded from further analysis. After the assessment, three types of data were collected. First, for each participant, responses labeled as “trochaic” and “iambic” were counted to examine whether there was any naming preference for the stress pattern in children with CIs. Due to the limited number of participants, we employed a binary logistic regression model to predict the stress pattern preference (trochee vs. iamb) based on the basis of two predictor variables, group (CI vs. NH) and item pattern (trochee vs. iamb). Second, the participants’ responses were coded as 1 (correct) or 0 (incorrect) to compare whether children with CIs produced one stress pattern better than the other. We conducted a binary logistic regression analysis to predict the correctness of naming responses (correct vs. incorrect) based on the basis of two predictor variables, i.e., group (CI vs. NH) and item pattern (trochee vs. iamb). Third, for novel words articulated with correct stress patterns, each response was annotated and manually checked. We examined the F0 range and duration of syllables were examined using Praat version 6.4.01 (Boersma & Weenink, 2023). The two acoustic measurements were selected because previous studies reported that Mandarin-speaking children with CIs generally exhibited a smaller F0 range for lexical tones and longer duration for the neutral tone (e.g., Tang et al., 2019). F0 range was measured as the difference between the maximal and minimal values for each syllable within a correct response. We were interested in the characteristics of stress patterns produced by children with CIs. Thus, for each stress pattern, we performed analysis using linear mixed-effects regression models with group (CI vs. NH) and syllable status (stressed vs. unstressed) as fixed factors, and with item and participant as random factors.

For the referent-matching task, we examined whether the children with CIs could match the novel word with its referent as well as their NH peers, and whether these children performed better when identifying words with a particular stress pattern. Thus, a binary logistic regression analysis was conducted. The model included two predictors: group (CI vs. NH) and item pattern (trochee vs. iamb). The dependent variable was the correctness of the response (coded as 1 for a correct response and 0 for an incorrect response).

For both the logistic and linear mixed-effects models, the significance of the predictor variables was assessed using Likelihood Ratio Tests. These statistical analyses were carried out with the package *lme4* (Bates et al., 2015) in R version 4.4.1 (R Development Core Team, 2024), and all effects were reported as significant at *p* < 0.05.

## 3. Results

All the participants were able to follow the experimental directions and provided responses. Among the 960 naming responses (16 disyllables × 2 stress patterns × 15 participants × 2 groups), 5 responses were excluded from the CI group, and 2 responses were excluded from the NH group, because they were either monosyllabic or trisyllabic. In addition, 13 responses were excluded from the CI group and 15 responses were excluded from the NH group, because they received inconsistent judgments from the three native Mandarin-speaking adults.

### 3.1. Quantity of Stress Pattern and Accuracy in Referent Naming

**Figure 2:**
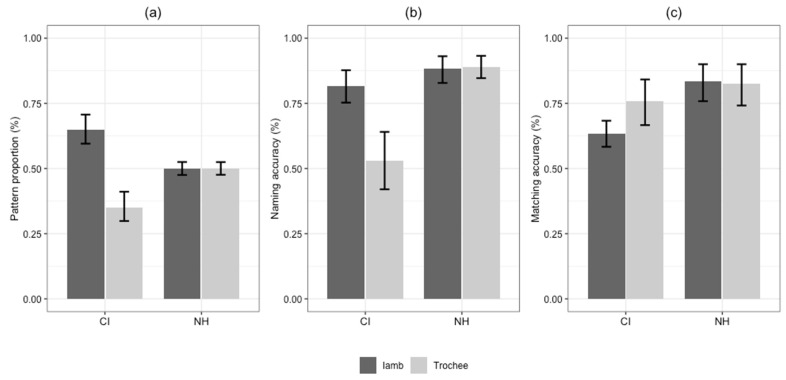
Results from referent-naming and referent-matching tasks in novel word learning paradigm for children with cochlear implants (CI) and normal hearing (NH). (**a**) Proportion of stress patterns produced. (**b**) Mean naming accuracy score by stress pattern of novel word. (**c**) Mean matching accuracy score by stress pattern of novel word. Error bars represent the 95% confidence intervals. Note: Stress patterns were experimentally assigned to novel word stimuli to examine prosodic effect and do not represent lexically contrastive patterns in Mandarin.

We first investigated whether there was any preference for trochees or iambs in referent naming, regardless of response accuracy. Figure 2a presents the comparison between the two groups in the proportion of stress patterns produced. Overall, the CI group produced more iambic words than trochaic words, whereas the NH group showed no obvious preference. Statistically, the main effect of group was significant, *b* = −2.24, *SE* = 0.33, *z* = −6.78, *p* < 0.001, *OR* = 0.11, 95% CI [0.05, 0.20], indicating that for the experimentally constructed trochaic items, the NH group had significantly lower odds than the CI group of producing iambic responses. The main effect of item pattern was also significant, *b* = 2.45, *SE* = 0.27, *z* = 9.21, *p* < 0.001, *OR* = 11.57, 95% CI [6.99, 19.88], suggesting an overall tendency toward iambic responses. More importantly, the interaction between group and item pattern was significant, *b* = 3.21, *SE* = 0.48, *z* = 6.64, *p* < 0.001, *OR* = 24.77, 95% CI [9.88, 66.18]. Follow-up simple effect analyses revealed that, for trochaic novel words, the CI group was more likely to produce iambic responses than the NH group (*p* < 0.001, *OR* = 9.40). For iambic novel words, the CI group was less likely to produce iambic patterns (*p* = 0.017, *OR* = 0.38).

For responses assessed as correct, we analyzed whether participants exhibited a production advantage for trochees or iambs. Figure 2b presents response accuracy for each experimental condition in the naming task. The analysis of accuracy revealed a significant main effect of group, *b* = 2.13, *SE* = 0.37, *z* = 5.78, *p* < 0.001, *OR* = 8.38, 95% CI [4.08, 17.23], indicating significantly higher naming accuracy in the NH group than in the CI group. A significant main effect of item pattern was also found, *b* = 1.51, *SE* = 0.23, *z* = 6.59, *p* < 0.001, *OR* = 4.51, 95% CI [2.88, 7.05], suggesting that participants produced significantly more correct responses for iambic novel words than for trochaic ones. In addition, a significant group × item pattern interaction was observed, *b* = −1.58, *SE* = 0.37, *z* = −4.24, *p* < 0.001, *OR* = 0.21, 95% CI [0.10, 0.43]. For the CI group, naming accuracy was significantly higher for iambic words than for trochaic words (*p* < 0.001, *OR* = 0.22), whereas the NH group showed no such iambic advantage (*p* = 0.804, *OR* = 1.08). Moreover, while the CI group performed significantly below their NH peers on trochaic words (*p* < 0.001, *OR* = 0.12), the two groups showed comparable accuracy for iambic words (*p* = 0.150, *OR* = 0.58). These results suggest that low naming accuracy in the CI group primarily occurs with trochaic items, while they nearly matched the NH group in iambic items.

In the above statistical analyses, 63 responses labeled as “equal prominence” were excluded, as they were either irrelevant to stress preference or considered incorrect. However, it is worth noting that the frequency of “equal prominence” responses varied across groups and item patterns. Among the responses produced by the CI group, 22 for trochaic novel words and 16 for iambic ones were judged as having equal prominence. Among those produced by the NH group, 9 for trochaic novel words and 16 for iambic ones were not classified as trochees or iambs.

### 3.2. Accuracy in Referent Matching

Figure 2c presents response accuracy for each experimental condition in the referent matching task. A significant main effect of group was found, *b* = 1.72, *SE* = 0.70, *z* = 2.46, *p* = 0.014, *OR* = 5.58, 95% CI [1.44, 22.58]. However, there was no significant main effect of item pattern, *b* = 1.25, *SE* = 0.67, *z* = 1.88, *p* = 0.06, *OR* = 3.50, 95% CI [0.96, 13.09], and no significant group × item pattern interaction, *b* = −0.66, *SE* = 0.45, *z* = −1.47, *p* = 0.142, *OR* = 0.52, 95% CI [0.22, 1.24]. These results suggest that the CI group was less likely than the NH group to correctly match novel words with their corresponding referents. By contrast, the stress pattern of the novel words did not affect referent matching performance in either group.

The absence of a significant interaction or item effect on matching accuracy indicates that, unlike the naming task where the CI group showed a strong iambic bias, their ability to recognize these words and match them to referents was not systematically biased by the stress pattern.

### 3.3. F0 Range and Duration of Syllables in Referent Naming

**Figure 3:**
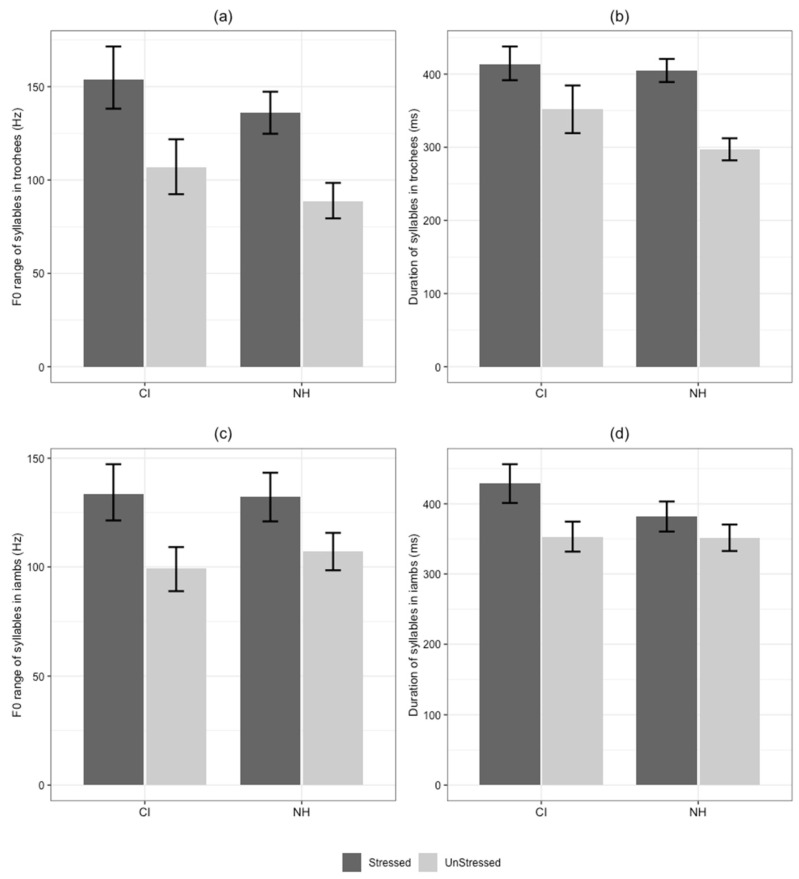
Comparison of acoustic properties between stressed and unstressed syllables produced in novel words by children with cochlear implants (CI) and normal hearing (NH). (**a**) F0 range in trochaic words. (**b**) Duration in trochaic words. (**c**) F0 range in iambic words. (**d**) Duration in iambic words. Error bars represent 95% confidence intervals. Note: Stress patterns were experimentally assigned to novel word stimuli to examine prosodic effect and do not represent lexically contrastive patterns in Mandarin.

Figure 3 shows the comparison of F0 range and duration of syllables in the two experimentally imposed stress patterns. Regarding the F0 range of trochaic novel words, the linear mixed-effects model revealed a significant main effect of syllable status, *b* = −47.36, *SE* = 9.96, *t*(604.1) = −4.75, *p* < 0.001, β = −0.271, 95% CI [−0.38, −0.16]. The non-standardized coefficient indicates that stressed syllables had a 47.36 Hz larger F0 range than unstressed syllables, and the standardized coefficient represents a medium effect of syllable status on modulation. Moreover, no significant main effect of group was found, *b* = −10.84, *SE* = 13.36, *t*(39.8) = −0.18, *p* = 0.422, β = −0.06, 95% CI [−0.21, 0.90]. Furthermore, the interaction between group and syllable status was also non-significant and characterized by a negligible effect size, *b* = −0.634, *SE* = 12.59, *t*(604.1) = −0.05, *p* = 0.96, β = −0.003, 95% CI [−0.14, 0.13]. These results collectively indicate that both NH and CI groups utilized F0 modulation as a primary cue to mark syllable stress in their novel word productions.

Regarding the F0 range of iambic novel words, only a main effect of stress status was found, *b* = −34.29, *SE* = 7.31, *t*(730.6) = −4.69, *p* < 0.001, indicating that stressed syllables had a 34.29 Hz larger F0 range than unstressed syllables. The standardized coefficient confirms a small-to-medium effect size for this acoustic contrast, β = −0.213, 95% CI [−0.30, −0.12]. However, no main effect of group was found, *b* = 1.87, *SE* = 11.66, *t*(40.3) = 0.16, *p* = 0.874, β = 0.012, 95% CI [−0.14, 0.16], nor was the group × stress status interaction, *b* = 8.57, *SE* = 10.16, *t*(730.6) = 0.84, *p* = 0.399, β = 0.047, 95% CI [−0.06, 0.16]. These negligible standardized effect sizes suggest that the contrast of F0 for iambic stress was comparable across the NH and CI groups, with no systematic differences in their phonetic realizations.

Regarding the duration of trochaic novel words, the analysis revealed a significant main effect of syllable status, *b* = −61.21, *SE* = 13.63, *t*(608.5) = −4.49, *p* < 0.001, β = −0.215, 95% CI [−0.31, −0.12], indicating that unstressed syllables were significantly shorter than stressed syllables. The main effect of group was not significant, *b* = −18.04, *SE* = 28.34, *t*(32.2) = −0.63, *p* = 0.536, β = −0.061, 95% CI [−0.26, 0.14]. More importantly, a significant interaction between group and syllable status was observed, *b* = −46.97, *SE* = 17.23, *t*(608.5) = −2.73, *p* = 0.007, β = −0.153, 95% CI [−0.26, −0.04]. This interaction indicates that the two groups differed in the magnitude of the duration contrast used to mark stress. Follow-up simple effect analyses revealed that while the two groups did not differ significantly in the duration of stressed syllables (18 ms, *p* = 0.536), the CI group produced significantly longer unstressed syllables than the NH group (65 ms, *p* = 0.031). In addition, while both groups shortened unstressed syllables relative to stressed ones, the duration contrast was substantially larger in the NH group (108.2 ms, *p* < 0.001) than in the CI group (61.2 ms, *p* < 0.001). These results suggest that although children with CIs utilize temporal cues to signal trochaic pattern, their implementation of this contrast is less robust than that of their NH peers.

Regarding the duration of iambic novel words, no main effect of group was observed, *b* = −46.29, *SE* = 24.65, *t*(34.9) = −1.88, *p* = 0.069, β = −0.144, 95% CI [−0.3, 0.01]; however, a significant effect of stress status was found, *b* = −76.51, *SE* = 11.77, *t*(732.7) = −6.5, *p* < 0.001, β = −0.239, 95% CI [−0.31, −0.17], as well as a significant group × stress status interaction, *b* = 47.03, *SE* = 16.36, *t*(732.7) = 2.87, *p* = 0.004, β = 0.129, 95% CI [0.04, 0.22]. The interaction indicates that the two groups implemented the duration contrast differently for iambic items. For stressed syllables, the CI group produced longer durations than the NH group, although this difference did not reach significance (46.3 ms, *p* = 0.069). For unstressed syllables, no significant group difference in duration was found (0.7 ms, *p* = 0.976). In addition, the duration difference between stressed and unstressed syllables was larger in the CI group (76.5 ms, *p* < 0.001) than in the NH group (29.5 ms, *p* = 0.01). These results suggest that in order to implement the iambic pattern, children with CIs tended to produce a larger contrast between stressed and unstressed syllables than their NH peers.

## 4. Discussion

The current study examined how Mandarin-speaking preschoolers with CIs learn novel words with overt trochaic and iambic prosodic patterns, compared to their NH peers. We hypothesized that children with CIs would show poorer word learning ability and different preferences for stress patterns when learning novel words with experimentally imposed stress patterns. The results revealed that children with CIs exhibited a distinct iambic advantage in naming tasks, both in terms of frequency and accuracy, whereas children with NH did not. In addition, although the CI group underperformed in the matching task, their recognition accuracy was unaffected by stress pattern.

The first question is why children with CIs showed an iambic advantage yet a trochaic challenge in referent naming. Our follow-up acoustic study provides a deep look into this phenomenon. Overall, stressed syllables had larger F0 ranges and longer durations than unstressed syllables. A significant between-group difference was observed in the duration of unstressed syllables within trochaic words. Specifically, children with CIs produced significantly longer unstressed syllables in these words, suggesting their difficulty in controlling temporal cues for trochees.

Two possible factors explain this difficulty in duration control. First, children with CIs struggle to shorten the second syllable in a trochee. As reported in previous studies, Mandarin-speaking preschoolers with NH often lengthen neutral-tone syllables (Zhu & Dodd, 2000), and they tend to produce durations exceeding those of adults (Tang et al., 2018). This lengthening pattern is even more pronounced in children with CIs (Shen et al., 2020; Tang et al., 2019; Yu et al., 2023). Children learn full tone lexical words with lengthened duration. They may not yet have mastered the shortened versions of these early-learned words. Although the current stimuli were novel words, they were constructed from familiar real words. For Mandarin speakers, a familiar syllable is typically associated with a canonical tone and stored as a full tone lexical entry. Inhibiting this ingrained full realization to produce a shortened version requires fine-grained motor control, which would be difficult for children with CIs (Adamidou et al., 2023). Second, lengthening the final syllable in an isolated word is cross-linguistically common. This phenomenon is referred to as final lengthening (Fletcher, 2010). Thus, if a stressed syllable requires longer duration, it is easier for children to produce an isolated iambic word, where the stressed syllable occurs in final position. Compared to their NH peers, our CI participants tended to produce longer second syllables in iambic words. In contrast, producing a trochee requires either lengthening the first syllable or shortening the second syllable. This poses greater challenges to children with CIs, who thus have more difficulty producing trochaic words than iambic ones.

Notably, final lengthening typically applies to prosodic domains above the word level (Paschen et al., 2022). According to the top-down model of prosodic development, children initially produce intonational or phonological phrases before producing syllables or feet (Santos & Fikkert, 2008). Thus, children with CIs may have produced the novel words along with the boundary cues of higher prosodic domains. Because isolated words in daily life are themselves intonational phrases, the boundary cue of final lengthening may influence word production (Pettinato et al., 2017; Snow, 1994; White, 2014). Our findings suggest that this factor influences children with CIs more than it does those with NH, who showed no naming bias toward trochaic or iambic words. Our current findings further suggest that the two groups of children may differ in prosodic processing. Children with CIs may over-generalize the lengthening patterns typically associated with higher prosodic domains and apply them to the word level, whereas children with NH are more likely to abstract the prosodic representation of novel words from various acoustic cues.

Although the “equal prominence” responses were excluded from the primary preference analysis, their occurrence is noteworthy. First, the higher frequency of these responses in the CI group, particularly for trochaic words, suggests their reduced ability to produce the duration contrast required for lexical stress. This flattened prosodic profile further reflects the challenges CI users face in phonetic implementation. Their failure to sufficiently shorten the unstressed syllable or lengthen the stressed syllable results in perceptually ambiguous stress patterns. Thus, these equal-prominence productions provide further evidence of prosodic deficits in children with CIs, specifically highlighting a failure to implement salient phonetic contrasts between syllables. Second, the stress patterns of participants’ naming responses were judged by three native Mandarin-speaking adults. Consequently, the equal-prominence judgments may be attributed to the inherent prosodic characteristics of Mandarin Chinese and the perceptual strategies of native listeners. Mandarin full-tone words lack salient acoustic and perceptual cues for word-level prominence. Our acoustic analyses revealed larger F0 ranges and longer durations of stressed syllables, highlighting the importance of F0 and duration cues in stress identification. As duration is a distinctive property of stress perception, it is possible that even if the participants attempted to implement a specific stress pattern, the acoustic cues might not have reached the threshold required for native listeners to perceive a clear stress contrast. Therefore, the equal-prominence responses stem from both the children’s difficulty in implementing robust phonetic contrasts and the listeners’ sensitivity to Mandarin’s predominantly balanced prosody.

The second question concerns why children with CIs showed no preference for either stress pattern when matching novel words with their corresponding referents. The results suggest that neither trochaic nor iambic patterns provide an advantage in forming lexical representations for Mandarin-speaking children. Although the prevalence of trochaic or iambic patterns in word learning may vary across languages and paradigms (Kehoe, 2018), evidence from nontonal languages shows that prosody plays a role in early word representation (Segal et al., 2020). Specifically, young language learners with NH form prosodically rich lexical representations of new words (Curtin et al., 2005). The current study extends this research to Mandarin Chinese, i.e., from languages with salient stress cues to one where such cues are largely absent. Mandarin full-tone words are much more common than those with neutral tones (W. Li, 1981). Although these words may theoretically exhibit a trochaic pattern (Duanmu, 2004; Hsieh, 2021), this pattern is difficult to perceive (Xu, 2023). As a consequence, Mandarin-speaking children, regardless of hearing status, may not be sensitive enough to use stress cues to form lexical representations. This aligns with recent findings in Greek-speaking children with CIs, who also showed no significant difference in identifying trochaic versus iambic words (Adamidou et al., 2023). Because Greek has no predominant stress pattern, children exhibit no lexical stress bias during referent matching. Similarly, our results support the view that when no salient stress pattern exists, no specific bias emerges in word learning. Although we experimentally imposed contrastive stress patterns, Mandarin-speaking children may not be sensitive to either pattern due to their native language background. Hence, stress pattern had no effect on word recognition.

The discrepancy between the observed iambic bias in production and its absence in recognition requires a cautious interpretation of these children’s underlying phonological representations. While children with CIs showed a clear iambic preference during naming, the lack of a corresponding effect in matching suggests that this bias may not be deeply rooted in their lexical representations. Instead, the production-only effect might stem from motoric or phonetic constraints. Specifically, children with CIs may face challenges in implementing the duration contrasts required for Mandarin trochees. Consequently, the iambic preference may reflect a distinction between phonetic performance and phonological representation. In this view, the overt output may be driven by articulatory constraints rather than by abstract phonological representations.

The finding that children with CIs had fewer correct responses than NH children suggests that they were not able to learn the novel words as well as their hearing peers. Children with profound hearing loss have deficits in word learning relative to their hearing peers (Houston et al., 2020). In early word acquisition, infants with NH form prosodically rich lexical representations of newly encountered words. The salient acoustic properties associated with lexical stress facilitate word-referent associative learning (Curtin, 2010). Children with CIs lack early exposure to oral speech and receive reduced-quality speech signals; hence, they have inadequate perception of acoustic cues. As a result, they have more difficulty perceiving contrastive lexical stress, face more challenges in building phonological representations of words, and demonstrate lower word learning ability.

On the other hand, our participants with CIs successfully discriminated between trochaic and iambic patterns and associated prosodic information with word meaning. This finding aligns with previous research showing that despite the degraded speech signals provided by CIs, users can still use available cues to differentiate stress patterns (Segal et al., 2016). Therefore, CIs devices still assist children’s language development (Sharma et al., 2020). Ultimately, our findings support the view that although children with CIs generally do not display linguistic skills equivalent to those of their NH peers, their language skills typically improve following implantation (Nittrouer et al., 2018).

Several limitations must be acknowledged and merit consideration when interpreting our findings. First, the current experiment included only 15 participants per group due to specific clinical criteria required. This limited sample size may have reduced statistical power to detect more subtle, higher-order interactions within our mixed-effects models. Consequently, while the primary effects of stress pattern and group were clear, generalizability of these findings to the broader population of children with CIs should be interpreted with caution. Future studies with larger sample sizes are necessary to confirm these patterns.

Second, while tonal combinations were strictly controlled in constructing novel words, individual syllables were drawn from highly familiar real words. This approach may have introduced unintended lexical or phonotactic biases. Specifically, despite being presented as novel entities, these words might have triggered partial activation of familiar phonological representations in children’s mental lexicons. This possibility suggests that children’s performance might reflect reliance on existing lexical knowledge rather than formation of independent novel representations. Future studies could address this by using less frequent syllable-tone combinations or by strictly controlling for neighborhood density of constituent syllables.

Finally, regarding the absence of a significant stress effect in referent matching, while we propose that this reflects the prosodic nature of Mandarin, certain methodological factors must also be considered as alternative explanations. First, limited exposure provided during the learning phase might not have been sufficient to allow for robust encoding of prosodic contrasts. Moreover, overall task difficulty may have played a role. With an average accuracy of approximately 80% for children with NH and even lower performance for those with CIs, the task likely required significant cognitive effort for both groups to successfully encode novel word-referent associations. Under these conditions, children may prioritize mapping between the phonological label and the object over the encoding of lexical stress. Consequently, any potential processing advantage provided by a specific stress pattern may remain secondary to the primary goal of referent identification, leading to a lack of observed bias in recognition. Future research with increased exposure and more varied task difficulty levels is needed to further isolate these variables.

## 5. Conclusions

The current study investigated how Mandarin-speaking children with CIs learn novel words with overt contrasting lexical stress patterns. In Mandarin, full-tone words lack salient acoustic and perceptual cues for word-level prominence, unless a neutral tone is present. Mandarin-speaking children with CIs have difficulty acquiring the neutral tone. This prosodic characteristic provides a unique opportunity to examine effects of overt stress patterns on word learning in Mandarin-speaking children with CIs. Our results demonstrated an iambic advantage in word production among children with CIs, while no significant stress bias was observed in word recognition. Their difficulty of duration control observed from the trochaic novel words indicates a challenge in syllable shortening compared to lengthening. These findings corroborate previous research on neutral tone acquisition and provide new evidence for the modality-specific nature of prosodic processing in this population. Regarding underlying mechanisms, we propose that the syllable lengthening strategy may be attributed to an overflow of features from higher-level prosodic domains onto the word domain, although the specific role of the prosodic hierarchy in shaping these articulatory patterns remains a subject for further empirical validation. Moreover, our results suggest that Mandarin-speaking children may not form prosodically rich lexical representations for new words in the same manner as speakers of nontonal languages. This lack of robust prosodic representation explains the absence of a stress bias in word recognition, supporting the influence of language-predominant patterns on word learning.

## Figures and Tables

**Table 1:** Auditory characteristics of children with cochlear implants.

ID	Gender	Age (Month)	Implanted Age (Month)	CI Experience (Month)	PTA Unaided (dB)	PTA with CI (dB)
CI-1	M	64.90	40.83	24.07	110	23
CI-2	M	61.23	40.93	20.30	120	26
CI-3	M	64.0	33.30	30.70	100	26
CI-4	M	60.47	41.10	19.37	110	23
CI-5	M	68.53	48.10	20.43	90	20
CI-6	M	51.53	27.47	24.07	120	23
CI-7	M	48.90	15.73	33.17	90	25
CI-8	M	52.23	15.00	37.67	120	29
CI-9	M	48.30	29.83	18.47	97	29
CI-10	M	50.07	22.73	27.33	115	20
CI-11	M	50.83	30.97	19.87	100	27
CI-12	M	49.9	25.77	24.20	91	20
CI-13	F	67.10	42.77	24.33	90	24
CI-14	F	70.37	52.07	18.30	90	25
CI-15	F	52.97	22.97	19.00	90	21

Note. CI = cochlear implant; PTA = pure-tone average.

**Table 2:** Sixteen combinations of tones in real words and novel words.

No.	Tone Combination	Real Word	Gloss	Novel Word	
				Trochee	Iamb
1	T1 + T1	xiang1 jiao1	“banana”	**tuo1** hua1	tuo1 **hua1**
2	T1 + T2	tuo1 xie2	“slipper”	**qian1** mei2	qian1 **mei2**
3	T1 + T3	qian1 bi3	“pencil”	**xiang1** gou3	xiang1 **gou3**
4	T1 + T4	ji1 dan4	“egg”	**ji1** shi4	ji1 **shi4**
5	T2 + T1	hong2 hua1	“red flower”	**hong2** jiao1	hong2 **jiao1**
6	T2 + T2	yuan2 xing2	“circle”	**ping2** qiu2	ping2 **qiu2**
7	T2 + T3	ping2 guo3	“apple”	**wan2** shou3	wan2 **shou3**
8	T2 + T4	wan2 ju4	“toy”	**yuan2** fan4	yuan2 **fan4**
9	T3 + T1	bing3 gan1	“cookie”	**xiao3** gan1	xiao3 **gan1**
10	T3 + T2	cao3 mei2	“strawberry”	**mi3** xing2	mi3 **xing2**
11	T3 + T3	xiao3 gou3	“poppy”	**bing3** bi3	bing3 **bi3**
12	T3 + T4	mi3 fan4	“rice”	**cao3** ju4	cao3 **ju4**
13	T4 + T1	mian4 bao1	“bread”	**dian4** bao1	dian4 **bao1**
14	T4 + T2	qi4 qiu2	“balloon”	**you4** xie2	you4 **xie2**
15	T4 + T3	you4 shou3	“right hand”	**mian4** guo3	mian4 **guo3**
16	T4 + T4	dian4 shi4	“television”	**qi4** dan4	qi4 **dan4**

Note. Bold face marks the stressed syllables in the novel words.

**Table 3:** Sixteen trails for learning phase, referent-naming task and referent-matching task.

ID	Learning and Presentation Sequence for Naming Task		Audio Recording for Matching Task
	First Learned	Second Learned	
1	tuo1 **hua1**	**tuo1** hua1	tuo1 **hua1**
2	qian1 **mei2**	**qian1** mei2	**qian1** mei2
3	**xiang1** gou3	xiang1 **gou3**	**xiang1** gou3
4	ji1 **shi4**	**ji1** shi4	ji1 **shi4**
5	**hong2** jiao1	hong2 **jiao1**	hong2 **jiao1**
6	**ping2** qiu2	ping2 **qiu2**	**ping2** qiu2
7	wan2 **shou3**	**wan2** shou3	**wan2** shou3
8	**yuan2** fan4	yuan2 **fan4**	yuan2 **fan4**
9	**xiao3** gan1	xiao3 **gan1**	**xiao3** gan1
10	mi3 **xing2**	**mi3** xing2	mi3 **xing2**
11	**bing3** bi3	bing3 **bi3**	bing3 **bi3**
12	cao3 **ju4**	**cao3** ju4	cao3 **ju4**
13	dian4 **bao1**	**dian4** bao1	**dian4** bao1
14	**you4** xie2	you4 **xie2**	you4 **xie2**
15	**mian4** guo3	mian4 **guo3**	**mian4** guo3
16	qi4 **dan4**	**qi4** dan4	**qi4** dan4

Note. Bold face marks the stressed syllables imposed in the novel words.

## Data Availability

The datasets analyzed during this study are available in the OSF repository, [https://osf.io/28vxn/overview?view_only=b845fd09efa6406a9c6fe195f1774319] (accessed on 29 January 2026).
